# TRIM21 Promotes cGAS and RIG-I Sensing of Viral Genomes during Infection by Antibody-Opsonized Virus

**DOI:** 10.1371/journal.ppat.1005253

**Published:** 2015-10-27

**Authors:** Ruth E. Watkinson, William A. McEwan, Jerry C. H. Tam, Marina Vaysburd, Leo C. James

**Affiliations:** Division of Protein and Nucleic Acid Chemistry, Medical Research Council Laboratory of Molecular Biology, Cambridge, United Kingdom; Mount Sinai School of Medicine, UNITED STATES

## Abstract

Encapsidation is a strategy almost universally employed by viruses to protect their genomes from degradation and from innate immune sensors. We show that TRIM21, which targets antibody-opsonized virions for proteasomal destruction, circumvents this protection, enabling the rapid detection and degradation of viral genomes before their replication. TRIM21 triggers an initial wave of cytokine transcription that is antibody, rather than pathogen, driven. This early response is augmented by a second transcriptional program, determined by the nature of the infecting virus. In this second response, TRIM21-induced exposure of the viral genome promotes sensing of DNA and RNA viruses by cGAS and RIG-I. This mechanism allows early detection of an infection event and drives an inflammatory response in mice within hours of viral challenge.

## Author Summary

Our cells have potent immune sensors that can detect the presence of viral nucleic acid in the cytosol. Unfortunately, almost all viruses utilize a strategy of encapsidation, comprising a protein shell that protects their genomes and impedes them from being sensed or degraded. In our study, we describe how components of innate and adaptive immunity combine to allow the rapid sensing of genomes from incoming viruses. We show that a ubiquitous immune protein called TRIM21 intercepts virions immediately after they enter the cytosol and exposes their genomes to nucleic acid sensors, thereby activating immune transcription pathways before genome replication commences. We demonstrate that TRIM21 enables the RNA sensor RIG-I to detect infection by an incoming RNA virus and the DNA sensor cGAS to detect infection by a DNA virus. By facilitating the sensing of incoming rather than progeny genomes, TRIM21 facilitates a rapid immune response upon infection. In the final part of our manuscript, we illustrate that this system confers an advantage to the host *in vivo* by demonstrating that there is a rapid TRIM21-dependent inflammatory response in mice upon viral infection, whereas in the absence of TRIM21 production of crucial cytokines like interferon is delayed.

## Introduction

TRIM21 is a ubiquitously expressed high-affinity cytosolic antibody receptor and E3 ubiquitin ligase [1]. TRIM21 intercepts incoming antibody-opsonized virions during cellular infection, mediating efficient post-entry neutralization [2] and innate immune signaling [3,4]. Unlike Fc gamma receptors, which phagocytose immune complexes, TRIM21 detects antibody-bound virions that enter the cytosol after attachment of the virus to its specific cellular receptor, endocytosis, and endosomal escape. TRIM21 therefore detects viruses during what could otherwise be a productive infectious event and protects cells of diverse tissue types [3]. TRIM21 activation does not require any pathogen associated molecular pattern (PAMPs) or pattern recognition receptors (PRRs) but is based solely on sensing antibodies in the cytosol, an environment from which they are normally excluded. Consequently, TRIM21 is activated during infection by diverse pathogens including non-enveloped viruses and intracellular bacteria [3]. TRIM21 participates in both naïve infection (through its ability to bind IgM) and secondary infection (by binding IgG). Upon in vivo challenge with mouse adenovirus 1 (MAV-1), mice lacking TRIM21 succumb to fatal viral infection within 7 days [5].

Upon detection of an antibody-coated virus in the cytosol, TRIM21 synthesizes K63 ubiquitin chains and activates the innate immune pathways NFκB, AP-1 and IRF3/5/7[3]. This leads to a broad program of antiviral transcription and induction of an anti-viral state. Concurrent with stimulating signaling, TRIM21 recruits p97/VCP, a AAA+ ATPase with segregase and unfoldase activity, and the proteasome, resulting in premature disassembly and degradation of viral capsids. This rapid degradation of incoming viral particles provides a potent block to infection [1,6]. We hypothesized that this catastrophic uncoating might also expose viral genomes for sensing by nucleic acid pattern recognition receptors (PRRs). Despite the presence of numerous cytosolic PRRs that recognize pathogen nucleic acids, it is often viral transcripts or progeny genomes, as opposed to incoming genomes, that are detected [7,8,9].

Here we show that TRIM21 potentiates the sensing of antibody-bound DNA or RNA viruses by cytosolic nucleic acid sensors cGAS or RIG-I. In addition, we identify that TRIM21-dependent innate immune signaling contributes a substantial component of the antibody block to rhinovirus infection in cultured cells, and demonstrate that TRIM21 and neutralizing antibody together drive a rapid pro-inflammatory transcriptional response upon adenovirus infection in mice.

## Results

### Antibodies potentiate immune activation during adenovirus or rhinovirus infection

**Fig 1:**
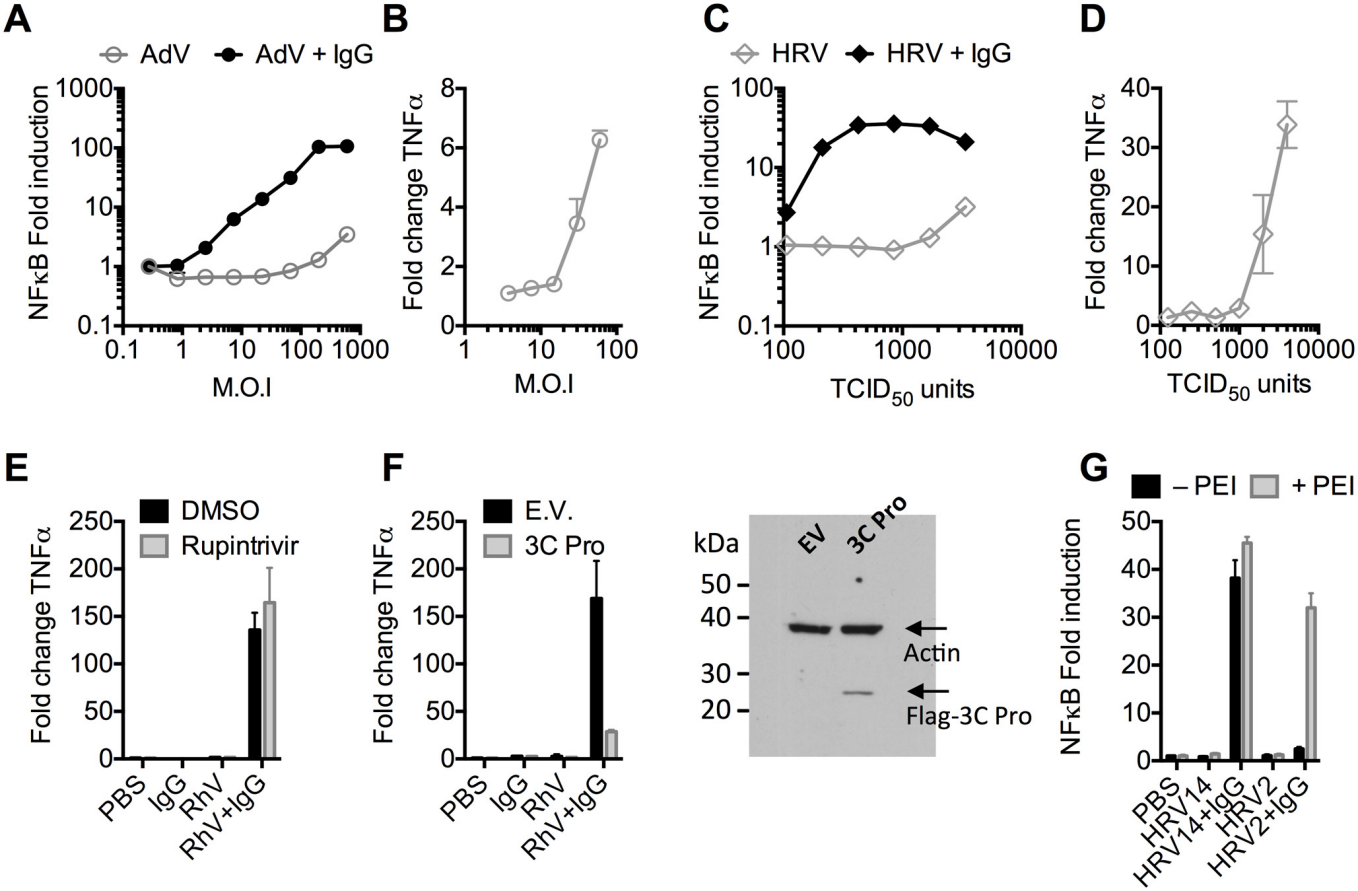
Antibodies potentiate immune sensing during adenovirus (AdV) or rhinovirus (HRV) infection. (**A**) Increase in NFκB-driven luciferase activity upon infection of MEF cells with AdV or AdV pre-incubated with human serum IgG. **(B)** TNFα mRNA levels in MEF cells 8 hours post challenge with AdV. **(C)** As (A), except using HRV. **(D)** As (B), except using HRV. **(E&F)** Induction of TNFα transcription in HRV infected cells treated with rupintrivir **(E)** or expressing 3C protease **(F)**. **(G)** NFκB activation in cells challenged with HRV14 or HRV2 in the presence or absence of PEI.

Recombinant human adenovirus type 5 (AdV) dose-dependently activates immune transduction pathways and this can be sufficient to upregulate transcription of cytokines and chemokines [10]. We tested whether immune activation is potentiated in the presence of antibody (IgG). Activation of an NFκB reporter was observed during infection of AdV+IgG at a multiplicity of infection (MOI) > 2, which increased until an MOI of 100 (Fig 1A). In contrast, NFκB activation during infection of AdV alone was only observed at an MOI >100, although induced TNFα transcription could be detected at slighter lower viral doses (Fig 1B). Similar antibody potentiation of NFκB activation was observed during infection by the picornavirus human rhinovirus type 14 (HRV), although both NFκB and TNFα could be observed at a high viral dose (Fig 1C and 1D). Picornaviruses antagonize immune sensing through expression of 3C protease, which cleaves immune signalling components [11,12]. Previously, we have shown that complement C3 promotes RhV sensing but is a substrate for 3C protease[13]. Treatment with rupintrivir, which inhibits cleavage, increases complement C3-dependent immune activation. In contrast, we found that rupintrivir did not alter IgG-dependent sensing (Fig 1E). To confirm that 3C protease is capable of suppressing immune activation in our experiments, we over-expressed the protease and found that IgG-mediated upregulation of TNFα was reduced (Fig 1F). Taken together, this suggests that IgG potentiates sensing of rhinovirus infection in a manner that is not antagonized by 3C protease. This is consistent with rapid TRIM21-dependent degradation of incoming antibody-coated virions prior to their replication.

Picornaviruses use diverse mechanisms to infect cells[14]. While the major group virus HRV14 is believed to disrupt endosomal membranes similar to AdV, leading to lysis and release of the virion into the cytosol, others like the minor group virus HRV2 are thought to form a pore through which only the genome is passed[14]. To test whether the observed antibody-dependent enhancement of immune activation requires delivery of antibody-opsonized virions to the cytosol we compared NFκB activation during HRV2 and HRV14 infection. While HRV14 induced a robust NFκB response in the presence of antibody, HRV2 did not (Fig 1G). Importantly, immune activation was observed when HRV2+IgG was transfected into cells using PEI (polyethylenimine), an endosomal disruptor[15], supporting the hypothesis that exposure to the cytosol is a key determinant of sensing in these experiments.

### TRIM21 promotes two distinct waves of innate immune signaling in response to antibody-opsonized virus

**Fig 2:**
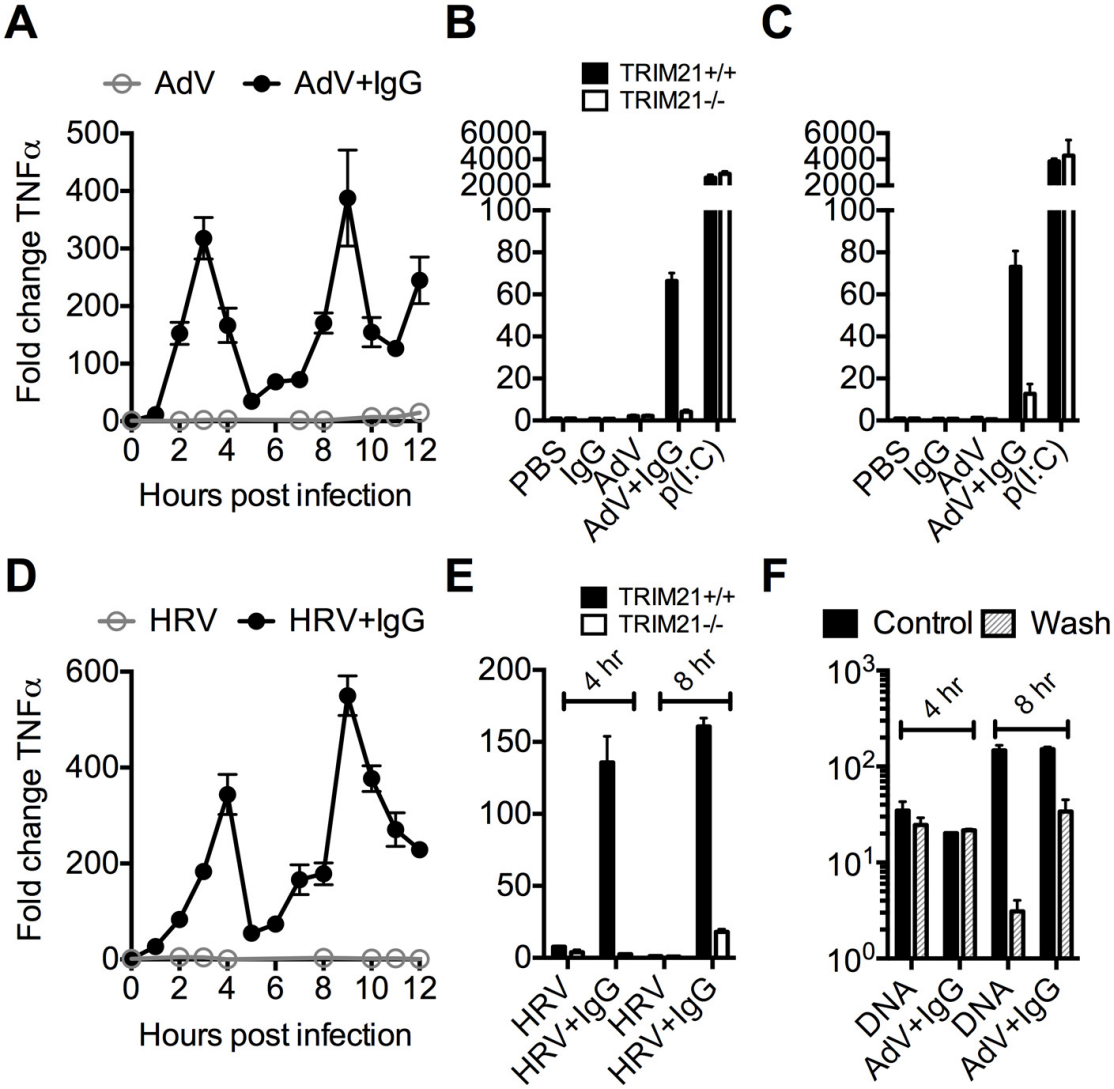
TRIM21 promotes two waves of innate immune signaling in response to antibody-opsonized virus. (**A**) TNFα mRNA levels in MEF cells following challenge with AdV or AdV pre-incubated with human serum IgG (AdV+IgG). (**B**) TNFα mRNA levels in MEF cells 4 hours post challenge with PBS, IgG, AdV, AdV+IgG, p(I:C). (**C**) As (B) except 8 hours. (**D**) TNFα mRNA levels in MEF cells following challenge with HRV or HRV+IgG. (**E**) TNFα mRNA levels in MEF cells after HRV or HRV+IgG. (**F**) TNFα mRNA levels in MEF cells challenged with DNA or AdV+IgG at 4 hours and 8 hours. Media either continuously exchanged (Wash, gray striped) or cells incubated as previously (Control, black).

To investigate the kinetics of antibody and TRIM21 dependent immune transcription, mouse embryonic fibroblasts (MEF) cells were challenged with AdV or HRV at a viral dose where no immune activation is observed in the absence of antibody (an MOI of 40 or TCID_50_ of 250, respectively). In the presence of antibody, infection provoked two sequential waves of TNFα transcription (Fig 2A and 2D). Comparison with TRIM21-/- MEF cells showed that TRIM21 is required for both the earlier (4 hours, Fig 2B and 2E) and later (8 hours, Fig 2C and 2E) responses, but not for TNFα transcription induced by transfected poly(I:C). We confirmed that, like for adenovirus, genetic deletion of TRIM21 had no impact on HRV infection or replication *per se*, but that TRIM21 was required for efficient neutralization of HRV (S1A–S1C Fig). Continuous media exchange to remove secreted cytokines from the cell supernatant had relatively little impact on TNFα induced by AdV+IgG compared to transfected DNA (Fig 2F), suggesting TRIM21-dependent signaling at 8 hours is the result of a second, independent, sensing event.

**Fig 3:**
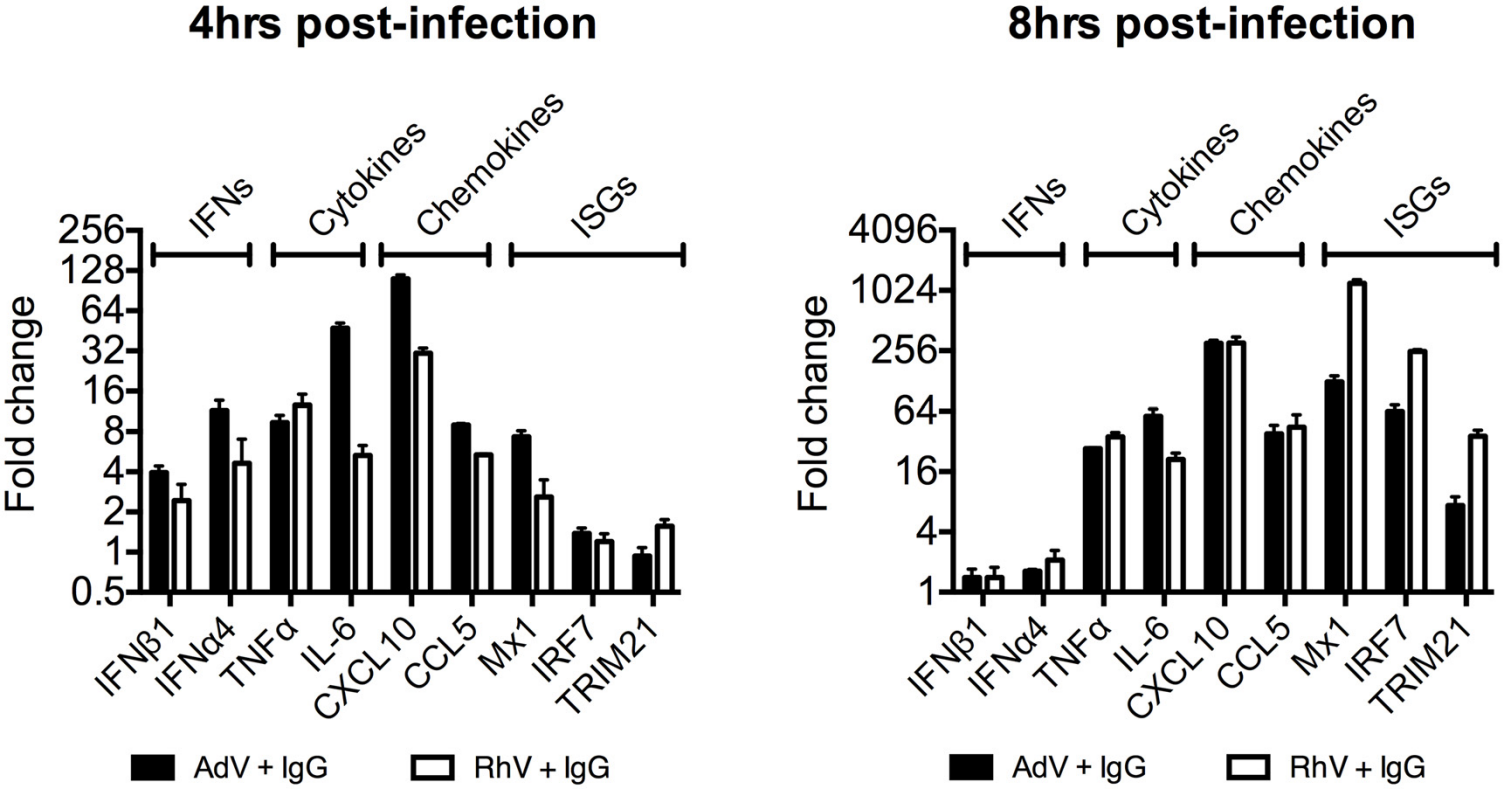
Transcriptional profiles induced by different antibody-opsonized viruses are differentially modulated during infection. Transcript levels of immune-related genes at 4 and 8 hours post-infection expressed as a fold change (AdV+IgG relative to AdV alone or HRV+IgG relative to HRV alone). Infection was carried out at an MOI of 20 (AdV) or TCID_50_ of 125 (HRV).

Comparing a panel of immune transcripts induced by AdV+IgG or HRV+IgG supports the interpretation that the second wave of signalling is not just an amplification of the first wave. At 4 hours post infection, AdV+IgG stimulated transcription more potently than HRV+IgG whereas at 8 hours this pattern was not maintained (Fig 3). In fact, there was substantially greater transcription of ISGs 8 hours after infection with HRV+IgG compared to AdV+IgG, suggesting that additional sensing events have occurred that differ between the two viruses. There was little change in any transcript upon infection in the absence of antibody, consistent with the NFκB and TNFα induction data (Fig 1 and S2 Fig).

### Antibody-dependent catastrophic uncoating reveals PAMPs

**Fig 4:**
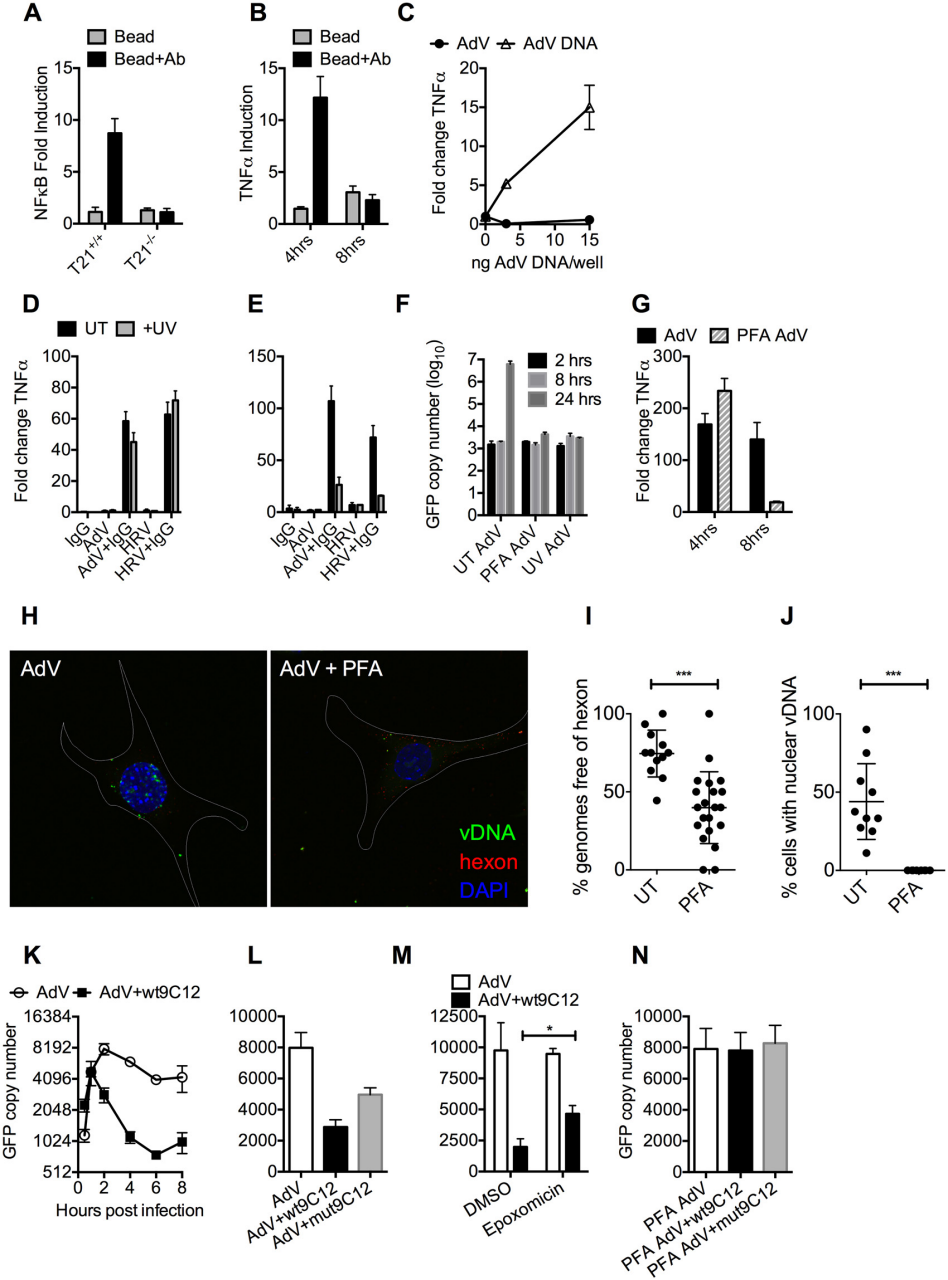
Antibody-dependent uncoating reveals viral genomes. (**A**) NFκB induction in TRIM21 +/+ and -/- MEFs upon transfection of IgG-coated beads. **(B)** As (A) except measuring the increase in TNFα mRNA at 4 and 8 hours post-infection in TRIM21 +/+ MEFs. **(C)** TNFα mRNA in MEF cells 8 hours post challenge with AdV (black squares) or transfected AdV DNA (white triangles). (**D**) TNFα mRNA levels in MEF cells 4 hours post challenge with IgG, AdV, AdV+IgG, HRV, or HRV+IgG (UT, black bars); or as above but following exposure of AdV and HRV to 500 mJ/cm^2^ UV irradiation (+UV, gray bars). (**E**) As (D) except 8 hours. (**F**) AdV genome copy number at various times post-infection with untreated (UT) or crosslinked (PFA or UV) virus. **(G)** TNFα mRNA levels in MEF cells treated with AdV+IgG relative to AdV alone (AdV) or AdV crosslinked with PFA pre-incubated with IgG relative to PFA-AdV alone (PFA-AdV). **(H)** Single-plane confocal micrographs of WT MEF cells 150 min post-infection with EdU-AdV. EdU-labelled vDNA is in green and anti-hexon 9C12 in red. White border delineates cell dimensions imaged by differential interference contrast. **(I)** Viral DNA puncta within each cell were scored for the presence of hexon labelling. **(J)** Fields of cells with >2 cells were scored for the presence of viral DNA in the nucleus. ***, P<0.0001 unpaired t-test. (**K**) AdV GFP copy number in MEF cells following infection with AdV (white circles) or AdV pre-incubated with 9C12 anti-AdV antibody (AdV+wt9C12, black squares). (**L**) AdV GFP copy number 2 hours post infection with AdV, AdV+wt9C12 or AdV pre-incubated with mutant 9C12 with reduced TRIM21 binding (AdV+mut9C12). (**M**) As (L), except cells pre-treated with proteasome inhibitor (epoxomicin) or solvent control (DMSO). * p < 0.05. (**N**) As (L), except PFA-crosslinked AdV.

We hypothesized that while the first wave of signaling may correspond to TRIM21 synthesis of K63-chain ubiquitin upon antibody binding, as previously described [3], the second wave may be the result of TRIM21-dependent exposure of viral PAMPs. To test this, we measured immune activation after transfection of cells with antibody-coated latex beads. Antibody-coated beads but not beads alone induced NFκB in a TRIM21-dependent manner (Fig 4A), consistent with previously published data[3]. Importantly, while antibody-coated beads activated TNFα transcription 4 hours post-transfection, there was no measurable response at 8 hours (Fig 4B). This is consistent with the second wave of sensing requiring a viral PAMP.

TRIM21 mediates rapid capsid degradation of incoming viruses, suggesting that the viral PAMP stimulating the second wave of transcription could be exposed viral genomes. Whilst we did not observe sensing of incoming AdV alone, equivalent concentrations of purified AdV DNA induced a TNFα response in transfected cells, suggesting that AdV DNA is stimulatory if exposed (Fig 4C). To provide evidence that the viral genome is the PAMP revealed by TRIM21 in the second sensing wave, we treated virus with ultraviolet (UV) irradiation to crosslink the nucleic acid. UV-crosslinking had no impact on antibody-dependent signaling at 4 hours post infection but TNFα transcription at 8 hours was substantially reduced (Fig 4D and 4E). Adenovirus is one of the most resistant pathogens to UV crosslinking[16], but comparison to untreated virus confirmed that irradiation had sufficiently modified the DNA to prevent it from undergoing replication (Fig 4F). Taken together, this suggests that unmodified genomes are required for the second TRIM21-dependent sensing wave.

Paraformaldehyde (PFA) is a chemical crosslinker that covalently couples protein-protein complexes together and has been used to stabilise virions and prevent their disassembly [17,18]. We hypothesised that if TRIM21-mediated virion degradation is required to expose the genome and provoke the second sensing wave then this should be inhibited by PFA-treatment. Consistent with this, PFA-crosslinking of adenovirus before incubation with antibody prevented TNFα transcription at 8 hours but not 4 hours post-infection (Fig 4G). To confirm that PFA crosslinking stabilises adenovirus virions we measured the impact of treatment on the uncoating of EdU-labelled virus. There was a significant decrease in the proportion of uncoated genomes (defined as dissociated from hexon) after PFA treatment (Fig 4H and 4I). Genome uncoating is a pre-requisite for nuclear entry and we also observed that PFA-crosslinking prevented import of vDNA into the nucleus (Fig 4J).

Kutluay et al. have previously shown that TRIM5α-mediated proteasomal degradation of incoming retroviral particles results in exposure of viral nucleic acid [19]. We used a similar approach to support our hypothesis that TRIM21-mediated proteasomal degradation also exposes viral genomes. Using qPCR to track incoming viral genomes, we noted that incubation with a non-entry blocking but TRIM21-dependent anti-adenovirus antibody (wt9C12) [2] did not substantially reduce adenovirus uptake (similar levels were observed at 1hr) but led to a marked reduction in AdV DNA after 2hrs (Fig 4K). Furthermore, 9C12 increased the rate of genome loss between 2–6 hours compared to infection by virus alone, a measure that is independent of how many genomes have entered the cell. The presence of nucleases in the cytosol capable of degrading exposed DNA was confirmed by incubating lysate with a FRET-labeled DNA probe (S3 Fig). Mutant antibody with reduced TRIM21 binding (mut9C12) or proteasome inhibition (epoxomicin) decreased genome loss but not to levels observed with virus alone (Fig 4L and 4M), consistent with previously published data showing that these perturbations reduce but do not abolish TRIM21 activity[1,2]. Importantly, while mut9C12 reduces TRIM21 binding it does not prevent binding to hexon (S4 Fig). To correlate the inhibitory effect of PFA-crosslinking on second wave sensing with prevention of genome exposure, we measured the impact of 9C12 on genome loss after infection with treated virus. Unlike with untreated virus, we observed no reduction in genome copies in the presence of wt9C12 (Fig 4N), suggesting that TRIM21 cannot mediate the efficient degradation of a PFA-crosslinked virion.

**Fig 5:**
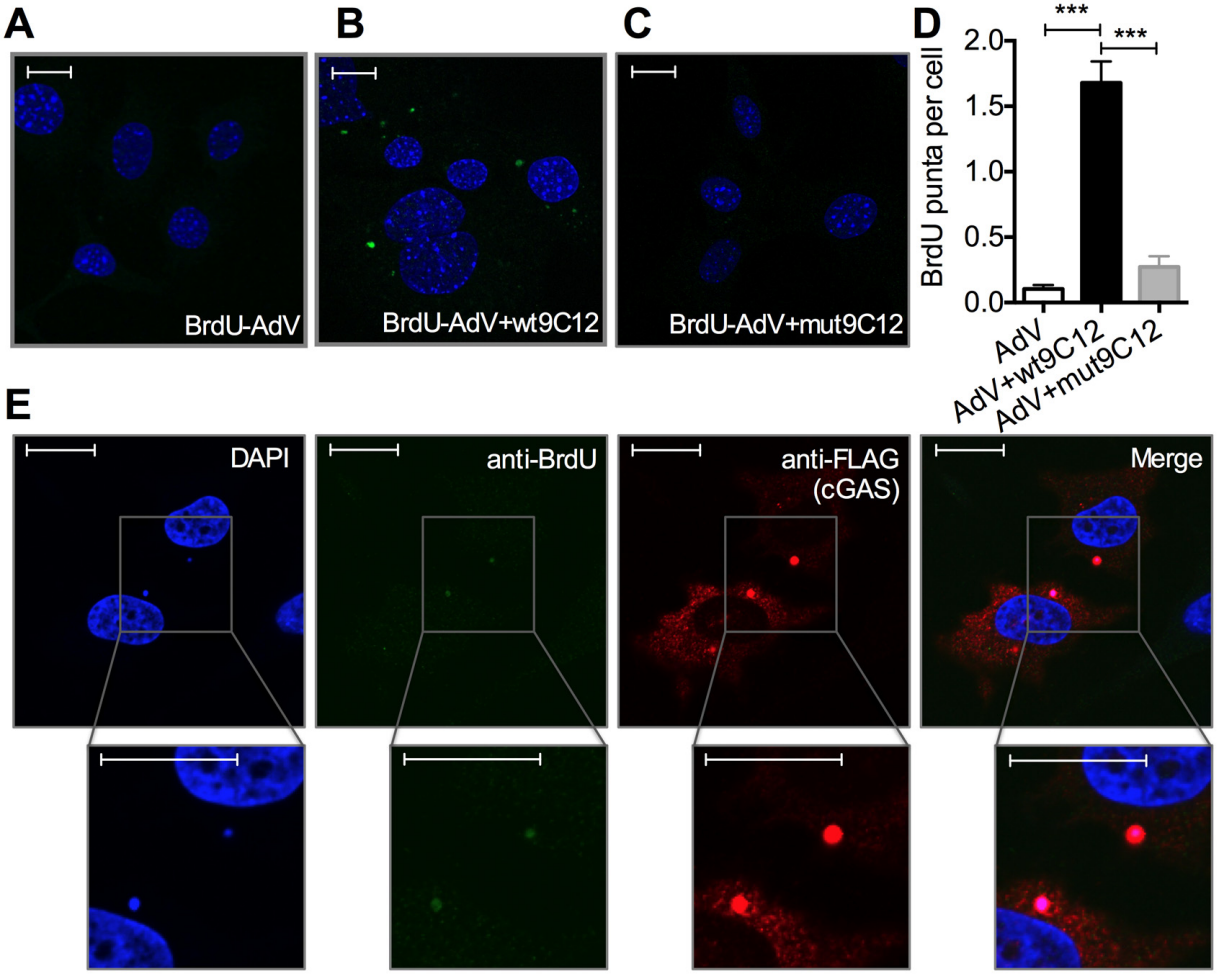
Antibody-opsonized virions have exposed genomes and colocalize with cGAS inside infected cells. (**A**) Confocal microscope image of MEF cells 2 hours post infection with AdV containing BrdU-labeled DNA (BrdU-AdV), stained with DAPI and anti-BrdU antibody. Scale bars show 20 μm. (**B**) As (A) except BrdU-AdV pre-incubated with wt9C12. (**C**) As (A), except BrdU-AdV pre-incubated with mut9C12. (**D**) BrdU-positive puncta per cell from confocal images from conditions G-I. *** p < 0.001, n ≥ 100 cells per condition. (**E**) As (B), except HeLa cells transfected with FLAG-cGAS prior to infection, and also stained with anti-FLAG antibody. Scale bars show 20 μm.

5-ethylyne-2’-deoxyuridin (EdU) in-situ labelling of the viral genome uses ‘click’ chemistry to install a small fluorescent probe on modified uracil nucleotides. It therefore may not distinguish uncoated nucleoprotein cores from naked vDNA, whose accessibility to DNA sensors may differ[20]. In contrast, bromodeoxyuridine (BrdU) labelling relies on the access of antibody to modified uracil bases and is therefore thought to be inhibited by proteins bound to viral DNA [21]. We used AdV with bromodeoxyuridine (BrdU)-labeled DNA (BrdU-AdV) and an anti-BrdU antibody to determine whether antibody-induced uncoating increases accessibility of the viral genome. While accessible AdV DNA was rarely detected following infection with BrdU-AdV alone (Fig 5A and 5D), pre-incubation of BrdU-AdV with wt9C12 antibody (Fig 4B and 4D), but not mut9C12 (Fig 5C and 5D), increased the appearance of BrdU-positive puncta. Furthermore, overexpressed FLAG-cGAS, a cytosolic DNA sensor, localized to these antibody-dependent BrdU-positive puncta (Fig 5E). The fact that far fewer virions were detected using BrdU labelling than in our EdU labelling experiments (Fig 4H) is consistent with the former requiring more accessible vDNA and may explain why adenovirus is poorly sensed in the absence of antibody even though uncoated genomes are present in the cytosol. Taken together, these data suggest that TRIM21-mediated capsid uncoating exposes the viral genome, allowing detection by cytosolic nucleic acid PRRs.

### TRIM21 and intracellular antibody promotes detection of viral nucleic acids by cytosolic nucleic acid sensors

**Fig 6:**
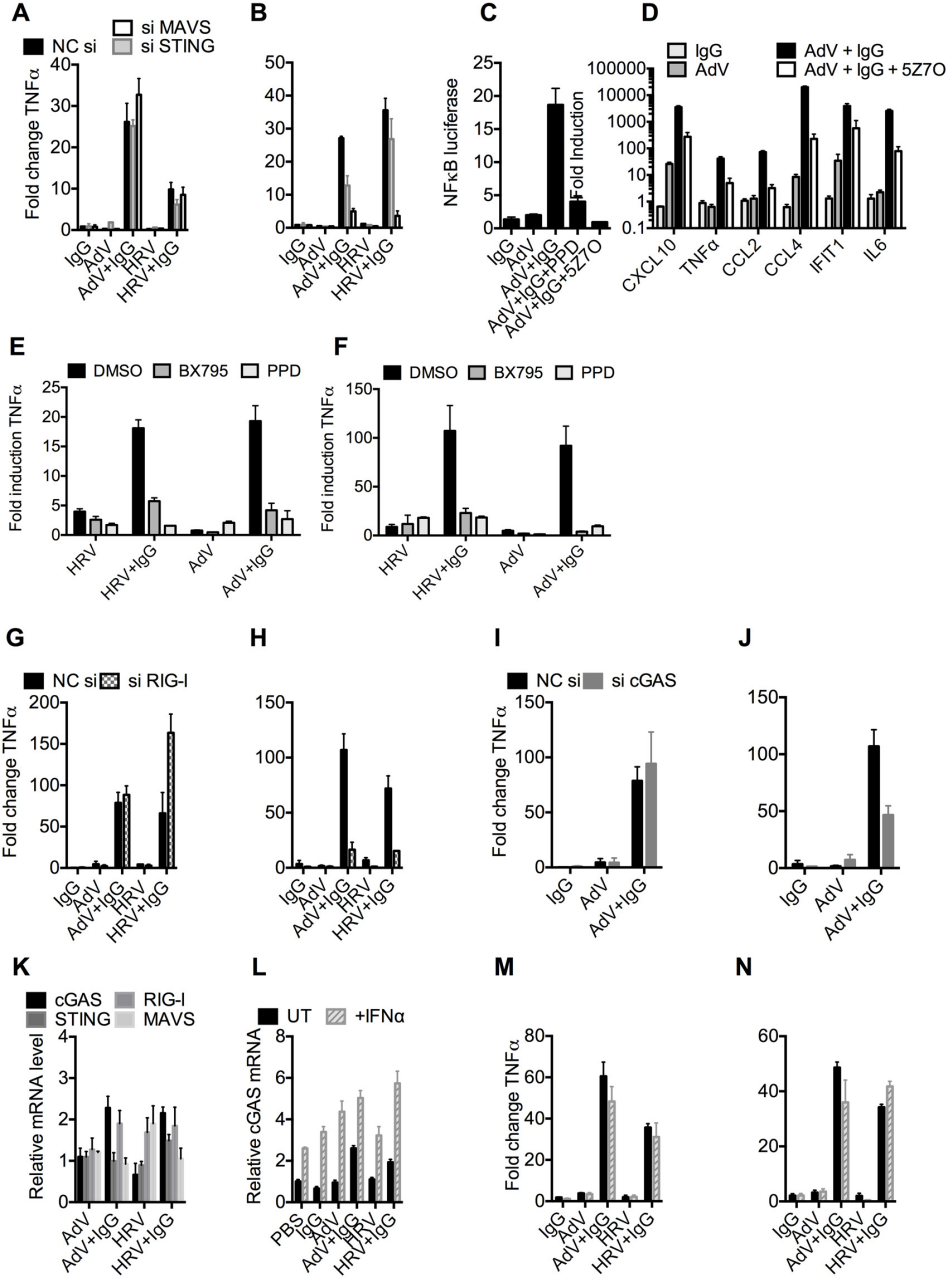
TRIM21 and antibody promote detection of viral nucleic acids by cytosolic sensors. (**A**) TNFα mRNA levels 4 hours post challenge of MEF cells with IgG, AdV, AdV pre-incubated with IgG (AdV+IgG), HRV, or HRV pre-incubated with IgG (HRV-IgG); MEF cells treated with negative control scrambled sequence siRNA (NC si, black), or siRNA against MAVS (siMAVS, white) or STING (siSTING, gray). (**B**) As (A) except 8 hours. (**C**) Activation of NFκB by AdV+IgG in the presence of panepoxydone (PPD) or 5*Z*-7-Oxozeaenol (5Z7O). **(D)** Induction of cytokine mRNAs 8 hours post-infection with AdV+IgG in the presence or absence of 5Z7O. **(E** and **F)** Induction of TNFα mRNA at 4hrs (E) or 8hrs (F) post-infection with HRV, HRV+IgG, Adv or Adv+IgG in the presence of PPD, TBK1 inhibitor BX795, or DMSO control. **(G)** TNFα mRNA levels 4 hours post challenge with IgG, AdV, AdV+IgG, HRV, or HRV+IgG; MEF cells treated with NC si (black), or siRNA directed against RIG-I (si RIG-I, gray checks). (**H**) As in (G) except 8 hours. (**I**) TNFα mRNA levels 4 hours post challenge with IgG, AdV or AdV+IgG; MEF cells treated with NC si (black), or siRNA directed against cGAS (si cGAS, gray). (**J**) As (I), except 8 hours. (**K**) cGAS, RIG-I, STING and MAVS mRNA levels in MEF cells 4 hours post challenge with AdV, AdV+IgG, HRV or HRV+IgG. (**L**) cGAS mRNA levels 4 hours post challenge with IgG, AdV, AdV+IgG, HRV or HRV+IgG on MEF cells either without (UT, black) or with simultaneous addition of recombinant IFNα (+IFNα, gray stripes). (**M**) TNFα mRNA levels from samples in (L). (**N**) TNFα mRNA levels 8 hours post challenge as in (M).

To test whether the genomes of incoming virions are sensed by nucleic acid PRRs in a TRIM21-dependent manner, we used siRNA to deplete MEF cells of STING or MAVS, the convergent adaptor proteins required for sensing cytosolic DNA and RNA, respectively[22] (S5A Fig). Depletion was confirmed by challenging cells with transfected DNA or poly(I:C) (S5B Fig). Neither STING nor MAVS depletion reduced antibody-dependent signaling 4 hours post infection with either AdV+IgG or HRV+IgG (Fig 6A), in agreement with previous results[3] and confirming that the first wave of sensing is primarily dependent on TRIM21 detection of antibody delivery to the cytosol. However, at 8 hours post infection, depletion of STING partially reduced TNFα transcription induced by AdV+IgG, while depletion of MAVS reduced transcription upon infection with either AdV+IgG or HRV+IgG (Fig 6B). These results are consistent with the known MAVS-dependent sensing of HRV RNA[23,24,25]; and detection of AdV by STING-dependent DNA sensors[10,26] and RNA polymerase III-dependent MAVS activation[7]. Although the later pathway involves host transcription of adenoviral DNA, it can occur prior to nuclear entry and productive infection, and in response to transfected poly(dA-dT) DNA, thus can still be considered a mechanism of detection of incoming nucleic acids[7].

MAVS and STING have been shown to transduce signals down the TBK1/IRF3 and IKK/NFkB [27,28] pathways. To investigate the involvement of these pathways in detection of AdV+Ab we used 5*Z*-7-Oxozeaenol, which inhibits TAK1, panepoxydone, which prevents IκB degradation, and the TBK1 inhibitor BX795, which blocks the phosphorylation and nuclear translocation of IRF3[29]. 5*Z*-7-Oxozeaenol and panepoxydone both inhibited NFκB induction 7 hours post-infection with AdV+Ab (Fig 6C). Moreover, NFκB inhibition had a direct effect on AdV+Ab-induced cytokine transcription. Transcripts of CXCL10, TNFα, CCL2, CCL4, IFIT1 & IL6 during the second wave were all reduced by either NFκB inhibitor (Fig 6D). Addition of BX795 inhibited both the first and second waves of TNFα transcription comparably with panepoxydone (Fig 6E and 6F). Together, this data suggests that immune activation induced by infection in the presence of antibody utilizes canonical transduction pathways known to be downstream of MAVS and STING.

Upstream receptors that activate MAVS and STING include the RNA sensor RIG-I and the DNA sensor cGAS. Consistent with the MAVS knockdown, depletion of RIG-I (S5C and S5D Fig) reduced TNFα activation by AdV+IgG and HRV+IgG at 8 hours (Fig 6H), but not 4 hours (Fig 6G). Meanwhile, siRNA depletion of cGAS (S5E and S5F Fig) inhibited detection of AdV+IgG 8 hours (Fig 6J), but not 4 hours, post infection (Fig 6I). Repeating our experiments using interferon priming to increase cGAS levels modestly beyond those under conditions of AdV+IgG or HRV+IgG infection at 4 hours post infection (Fig 6K and 6L) had no impact on sensing at either 4 hours (Fig 6M) or 8 hours (Fig 6N). Thus second wave sensing is not due simply to upregulated sensors but is consistent with antibody-enhanced detection of viral genomes.

### TRIM21 inhibits spreading infection and provides an early detection mechanism *in vivo*

**Fig 7:**
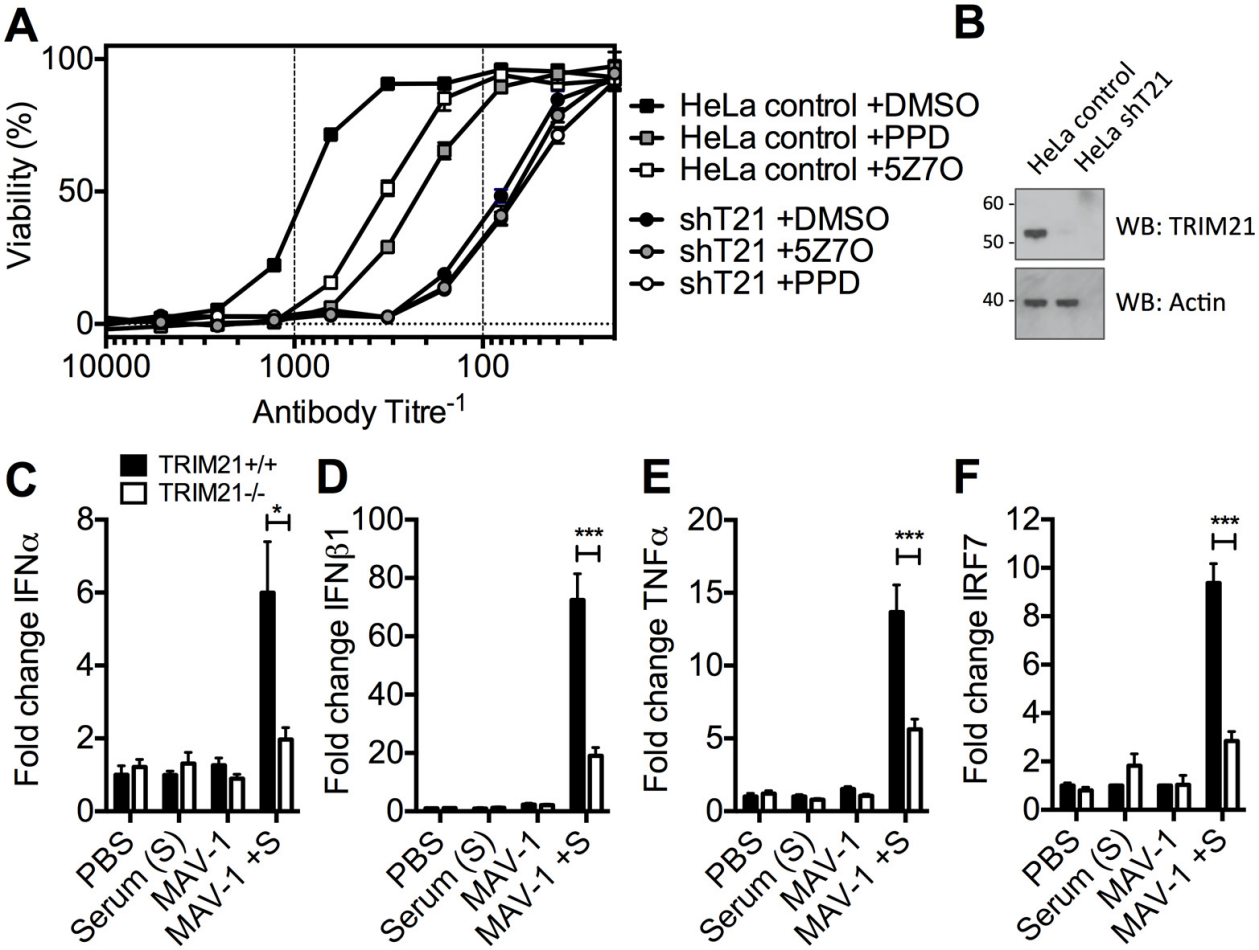
TRIM21-dependent signaling aids control of viral replication, and promotes inflammation *in vivo*. (**A**) Viability of HeLa cells or HeLa cells stably depleted of TRIM21 (shT21) following treatment with PPD or 5Z7O then infection with HRV pre-incubated with IgG. (**B**) Immunoblots for TRIM21 and β-Actin for cells as in (A). (**C**) IFNα mRNA levels in brain tissue 6 hours post infection, following passive transfer of immune serum (or PBS control) at day -1: PBS control plus mock infection (PBS); immune serum plus mock infection (Serum (S)); PBS control plus MAV-1 infection (MAV-1); immune serum plus MAV-1 infection (MAV-1 +S). (**D**) As (C) except IFNβ1 mRNA. (**E**) As (C) except TNFα mRNA. (**F**) As (C) except IRF7 mRNA. * p < 0.05 and *** p < 0.001.

To determine the physiological importance of TRIM21-mediated immune activation, we assessed its contribution to controlling HRV infection. In cells stably depleted of TRIM21, neutralization of HRV infection was substantially reduced (Fig 7A and 7B). Treatment of TRIM21-depleted cells with NFκB signaling pathway inhibitors, panepoxydone (PPD) and 5Z-7-oxozeaenol (5Z7O), gave no additional reduction in antibody protection. NFκB inhibition in control cells resulted in antibody protection intermediate between untreated control cells and TRIM21-depleted cells, suggesting that signaling forms part of the antiviral activity of TRIM21, with the remaining component due to TRIM21-dependent neutralization. Finally, we tested whether the ability of TRIM21 to promote immune activation prior to viral replication facilitates a rapid inflammatory response during infection *in vivo*. We infected TRIM21+/+ or TRIM21-/- mice with mouse adenovirus type 1 (MAV-1) by intraperitoneal injection in the presence or absence of anti-adenovirus immune serum (serum, S). A rapid inflammatory response was observed 6 hours post infection in the brain tissue of TRIM21+/+ animals that also received immune serum. In contrast, animals deficient in TRIM21 or lacking immune serum had significantly reduced or absent induction of IFNα (Fig 7C), IFNβ1 (Fig 7D), TNFα (Fig 7E) and the interferon stimulated gene IRF7 (Fig 7F).

## Discussion

The early detection of infection is advantageous to the host. However, most viruses are shielded by a protein capsid that protects their genomes and associated PAMPs from immune sensing. As a consequence, incoming viral genomes are not always detected by pattern recognition receptors and detection often requires progeny genomes produced at later stages of the infectious cycle [30]. For instance, the capsid of HIV-1 mediates a series of post-entry events that allows the virus to evade detection in macrophages by the cytosolic PRR cGAS[31,32]. Similarly, adenovirus infection proceeds via an elegant and highly coordinated stepwise process of disassembly[33], that may function in part to minimize exposure of the genome to DNA sensors in the cytoplasm. Here we show that TRIM21, which mediates the rapid degradation of incoming viral capsids in the cytosol, promotes the ability of sensors cGAS and RIG-I to detect the genomes of infectious viruses.

**Fig 8:**
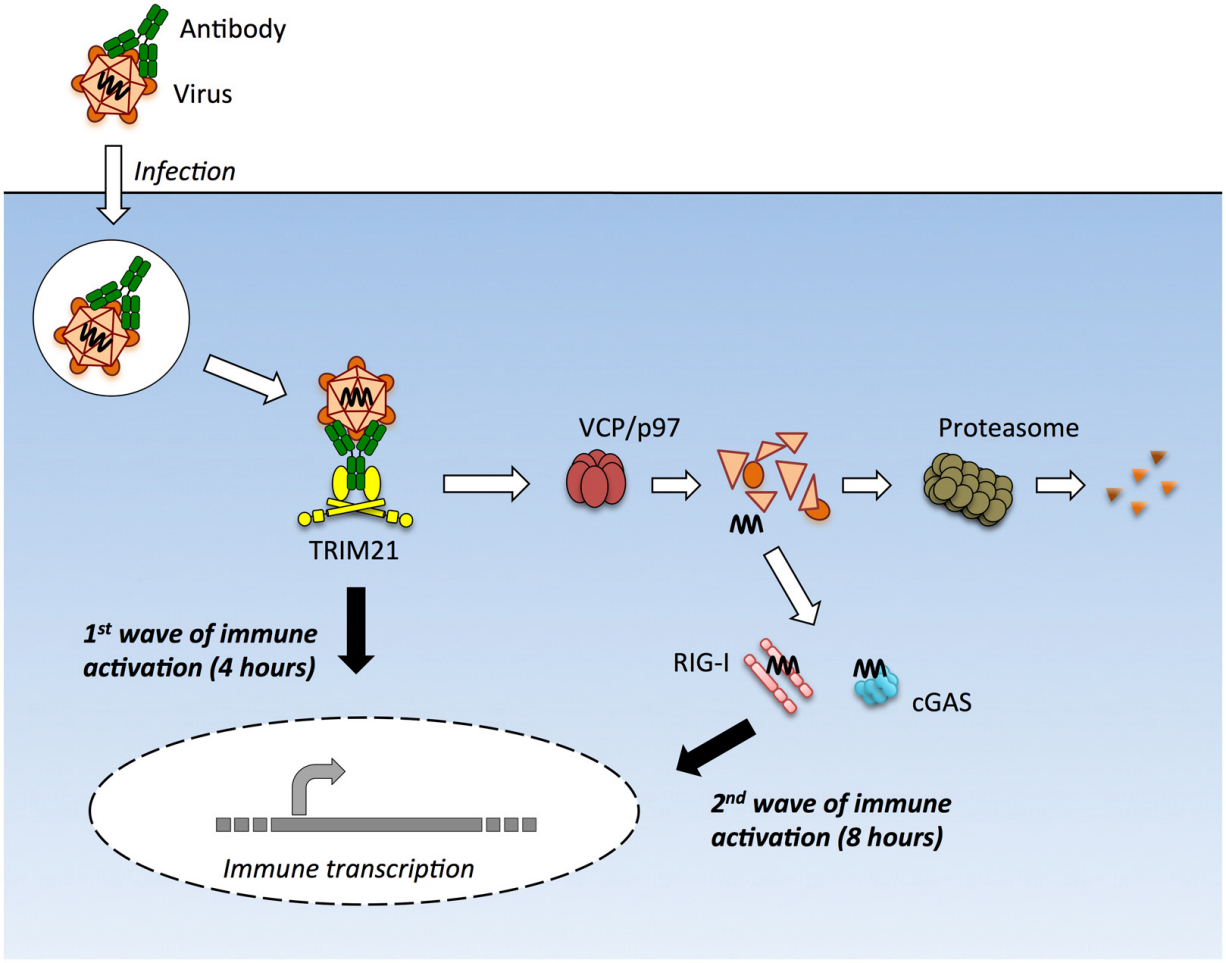
TRIM21 mediates multiple waves of immune sensing. TRIM21 intercepts antibody-bound virions during infection of cells of various hematopoietic or non-hematopoietic lineages, and mediates both innate immune sensing and post-entry neutralization. Upon recognition of intracellular antibody, TRIM21 catalyzes formation of K63-linked polyubiquitin chains, which directly activate components of the NFκB, AP-1 and IRF3/5/7 signaling pathways, resulting in transcription of type I IFN and other innate immune mediators at 4 hours post infection. Concurrent with signaling, TRIM21 recruits p97/VCP and the proteasome to promote premature catastrophic viral uncoating, and degradation of viral proteins. This both prevents productive infection, and exposes viral genomes from within viral capsids to the cytosol. Cytosolic PRRs including cGAS and RIG-I then detect these free viral nucleic acids, activating a second wave of immune transcription at 8 hours post infection.

Upon infection with either the DNA virus adenovirus or the RNA virus rhinovirus we observed two early waves of innate immune signaling, both of which were dependent on TRIM21. The first wave led to a relatively similar transcription pattern of immune-responsive genes for both viruses and did not require the adaptors MAVS or STING or the sensors RIG-I or cGAS. In contrast, the second wave of transcription was variously dependent upon these adaptors and sensors and led to a divergent enhancement of the response between viruses. In the absence of TRIM21, there was no significant immune induction within the first 8 hours of infection by either AdV or HRV. This was despite the fact that transfection of AdV vDNA provoked robust TNFα induction when assayed at 8 hours. Thus TRIM21 not only promotes the early detection of diverse viruses but also potentiates the activity of other PRRs to detect the highly immunostimulatory ligands that are shielded in an infectious viral particle (Fig 8).

Adenovirus virions can be sensed in the absence of antibody but at higher MOIs. Why adenovirus is normally poorly sensed is an important question, particularly given recent data showing that most uncoated viral genomes are not imported into the nucleus[21]. Our BrdU-labelling experiments suggest that although these genomes are dissociated from capsid, the vDNA is poorly accessible. This is consistent with the densely packed structure of the protein VII-containing adenovirus nucleoprotein core and its known insensitivity to nucleases and DNA repair enzymes[20,34]. Viral nucleoprotein complexes may be as important as the capsid shell in protecting incoming viral genomes from sensors.

The ability to intercept incoming virions allows TRIM21 to trigger an immune response during the early stages of cellular infection. We detected robust transcription of type I interferons, cytokines and ISGs 6 hours post infection in the brain tissue of wild-type but not TRIM21 mice challenged with MAV-1. An important aspect of TRIM21 function is that it depends on antibodies to detect virions in the cytosol. Consistent with this, we did not observe significant early immune activation in mice in the absence of immune serum. Importantly, these results identify a previously unrecognized component of antibody-mediated protective immunity *in vivo*. Furthermore, they provide an unusual example of adaptive immunity activating innate immunity, contrary to the classic progression from innate to adaptive responses via pro-inflammatory mediators. IgM antibodies of the primary repertoire, which TRIM21 can utilize, may be capable of inducing an early response during naïve infection, however we did not observe this during our experiments with MAV-1.

While TRIM21-mediated disassembly of infecting virions promotes both viral restriction and exposure of PAMPs, there may be competition between sensing and nuclease-mediated degradation of the genome. Indeed, this may contribute to the duration of TRIM21-dependent signaling waves. Whether genome degradation is a necessary step in viral restriction has not been fully investigated, but the premature uncoating of adenovirus may be sufficient to reduce infection by preventing microtubule engagement and trafficking of virions to the nucleus. This is supported by data showing that incubation of AdV with 9C12 inhibits localization of virions to the nuclear envelope [35].

TRIM21 degradation of incoming virus may be advantageous to sensing not only in revealing PAMPs but also in preventing the expression of viral antagonists of immune signaling. The sensing of progeny genomes is known to be highly susceptible to viral antagonism. For instance, influenza nonstructural protein 1 is a very effective RIG-I antagonist but is only produced upon replication [36]. The sensing of incoming virions and genomes by TRIM21, together with cGAS and RIG-I, may be harder for the virus to inhibit. In support of this, we found that TRIM21-dependent signaling is not antagonized by virus-produced HRV protease 3C but is inhibited if the protease is ectopically overexpressed prior to infection. Furthermore, TRIM21 potentiates the sensing of HRV by RIG-I, which does not normally contribute to picornavirus sensing because it is cleaved and rendered inactive by 3C protease [11]. Antagonism by 3C may be one explanation for the lack of immune detection of HRV in our experiments in the absence of TRIM21. TRIM21 may be facilitating sensing of HRV both because it exposes the incoming genome to RIG-I and because it degrades virions before 3C can be expressed.

Given the similarity between the dual functions of TRIM21 and those of TRIM5 as a retroviral capsid sensor and restriction factor[37,38,39], our findings suggest that TRIM5 may also promote detection of retroviral infection by revealing the incoming genome or early reverse transcription products from within the viral capsid. More generally, this study highlights the ability of PRRs to act synergistically. This may be advantageous to the host, both in amplifying immunity, and in guiding the resulting response to best counter the invading pathogen.

## Material and Methods

### Cells

TRIM21+/+ and TRIM21-/- MEF cell lines were generated as described previously^1^. HeLa cells are a human epithelial cervix carcinoma cell line (ATCC, Manassas, VA). Cells were maintained in Dulbecco’s modified eagle medium (DMEM) supplemented with 10% fetal bovine serum (FBS) and 100 U/ml penicillin and 100 μg/ml streptomycin. All cells were incubated at 37°C in the presence of 5% CO_2_, except where stated.

Knockdown by siRNA: MEF cells were transfected with siRNA using RNAiMAX (Life Technologies, Carlsbad, CA), and were incubated for 2 days before use in assays. siRNA as follows: negative control (Ambion, Carlsbad, CA), STING (Sc-154411, Santa Cruz, Dallas, TX), MAVS (s105943, Ambion), cGAS (1:1 mixture of duplexes with the following sequence: GAUUGAGCUACAAGAAUAU, GAGGAAAUCCGCUGAGUCA, Sigma), RIG-I (L-065328, Dharmacon, Lafayette, CO). Knockdown by shRNA: cells were transduced with retroviral particles encoding small hairpin RNA (shRNA) against human TRIM21 (GCAGCACGCTTGACAATGA) as described previously [2].

### Viruses

Human adenovirus type 5 vector (ΔE1,ΔE3) expressing GFP (AdV) was purchased from ViraQuest (North Liberty, IA). EdU-AdV and BrdU-AdV was produced by infection of the transcomplementation cell line HEK293T in the presence of 7.5 μM 5’-ethynyl-2’-deoxyuridine (EdU) or 5 μg/ml BrdU respectively (Sigma, St. Louis, MO). PFA-AdV was produced by incubation of AdV on ice for 3 hours in 4% PFA, followed by quenching with 0.1 M glycine, then dialysis to AdV storage buffer (25 mM Tris pH 8, 0.5 M NaCl). AdV and HRV were UV-crosslinked by exposure to 500 mJ/cm^2^ UV irradiation using a UV Stratalinker 2400 (Stratagene, La Jolla, CA). Human rhinovirus type 2 & 14 (HRV) were produced by infection of HeLa cells, and purified by 2 rounds of CsCl centrifugation as described previously [1]. Mouse adenovirus type 1 (MAV-1) (ATCC) was produced by infection of a 3T3 MEF cell line (ATCC), and virus was purified by 2 rounds of CsCl centrifugation as above.

### Viral infections to measure titer

HRV replication: 5 x 10^3^ cells were plated in 96 well plates overnight in DMEM supplemented with 2% FCS (low-serum media). Where stated, recombinant human IFNα (Sigma) was added to cells at the time of plating. 20 TCID_50_ units HRV was mixed 1:1 with human serum IgG (Sigma) (or PBS where no IgG was added) for 1 hour then added to cells. Cells were incubated for 6 hours, then washed and incubated in fresh low-serum media at 35°C for 7 days. Viability was then measured using 500 μg/ml MTT (3-(4,5- Dimethylthiazol-2-yl)-2,5-Diphenyltetrazolium Bromide) reagent (Sigma), with absorbance measurement at 540/650 nm using a SpectraMAX 340PC (Molecular Devices, Sunnyvale, CA).

AdV, UV-crosslinked AdV and PFA-crosslinked AdV were titrated onto HeLa cells (5 x 10^4^ cells per well plated overnight) then incubated for 24 hours. The percentage of cells positive for GFP expression was determined by fluorescence activated cell sorting (FACS) analysis using a BD LSRII machine.

AdV neutralization: 3.75 x 10^4^ IU AdV per well was mixed 1:1 with human serum IgG (IgG) at the stated concentration then added to HeLa cells (5 x 10^4^ cells per well plated overnight, MOI = 0.3) and incubated for 24 hours, then the percentage of cells positive for GFP expression was determined as above.

### Mice

C57BL6 wild type (TRIM21+/+) and TRIM21-/- mice used in all *in vivo* experiments were obtained from Jackson Laboratories (Bar Harbor, ME). All mice used in experiments were between 7 to 10 weeks old at the start of infection protocols. All experiments were conducted in accordance with the 19.b.7 moderate severity limit protocol and the Home Office Animals (Scientific Procedures) Act (1986). TRIM21+/+ and TRIM21-/- mice were infected by IP injection with 3.12 x 10^6^ TCID50 units MAV-1 in 100 μl PBS. MAV-1 antiserum was prepared from blood collected from C57BL/6 mice 72 days post infection following 3 rounds of immunization with a sub-lethal dose of MAV-1 as described previously [5]. Mice were injected by IP with 200 μl serum diluted 1:1000. In all cases, control mice received injection of the same volume of PBS buffer. At the end point of the experiment, brains were extracted and snap-frozen in liquid nitrogen.

### Cytokine analysis by qPCR from cultured cells

TRIM21+/+ and TRIM21-/- MEF cells were plated at a density of 1–2 x 10^4^ per well in 96-well plates overnight prior to infection. Viruses were mixed 1:1 with antibody and incubated for 30 mins at room temperature prior to infection. AdV was used at 7.5 x 10^5^ IU per well, HRV was used at 2.5 x 10^2^ TCID_50_ units per well unless otherwise stated. Human serum IgG (IgG) was used at 2.5 mg/ml. Where stated, human IFNα (0.1 U per well, Sigma) was added at the time of infection. p(I:C) (1 μg/ml, Sigma), herring sperm DNA (10 μg/ml, Life Technologies) and p(dA-dT) (10 μg/ml Sigma), were all transfected using lipofectamine 2000 (Life Technologies). AdV DNA was purified from virions using a DNeasy mini kit (Qiagen, Manchester, UK), and also transfected using lipofectamine 2000. Purified AdV DNA concentration and DNA concentration in AdV virions (following 10 mins incubation in 0.1% SDS detergent to disassemble virions) were determined using a NanoDrop 2000c (Thermo Scientific, Loughborough, UK), so that the equivalent concentration of total AdV DNA could be applied to cells by either infection or transfection. In all experiments using transfection of nucleic acids, results are normalized to mock transfection containing lipofectamine 2000 but no nucleic acids (however, this gave no significant signaling above baseline). Where indicated, cells were washed with PBS 1 hour post stimulation (infection or transfection of nucleic acid), then cells were either incubated in fresh media for the remaining time or media was exchanged every 15 mins from this point until the end of the experiment. At the stated end timepoints, cells were washed with PBS, then cDNA was prepared using Taqman Cells-to-CT (Fast) kit (Life Technologies). Relative gene expression compared to TBP reference gene was determined using the change-in-threshold (2^−ΔΔCT^) method, using Taqman gene expression assays (Life Technologies) on a StepOnePlus Real Time PCR machine (Applied Biosystems, Waltham, MA). Taqman gene expression assay mixes as follows: TATA-Box binding protein (TBP) Mm00446971_m1, Tumor necrosis factor α (TNFα) Mm00443260_g1, Interferon α4 (IFNα4) Mm00833969_s1, Interferon β1 (IFNβ1) Mm00439552_s1, CXCL10 Mm00445235_m1, Interferon regulatory factor 7 (IRF7) Mm00516791_g1, CCL5 Mm01302427_m1, Myxovirus resistance 1 (Mx1) Mm00487796_m1, MAVS Mm00523170_m1, STING Mm01158117_m1, RIG-I Mm01216853_m1, cGAS Mm00557693_m1.

### qPCR quantification of AdV DNA in infected cells

MEF cells were plated at a density of 1 x 10^4^ cells per well in 96-well plates overnight before infection with AdV. AdV was used at 7.5 x 10^5^ infectious units (IU) per well mixed 1:1 with wt9C12 and mut9C12 (both 25 μg/ml, produced as described previously (McEwan et al. 2013)) in a total volume of 5 μl for 30 mins before addition to cells. 1 hour post infection, cells were washed with PBS x 3 then incubated in fresh media until the endpoint of the experiment. Where PFA-AdV was used, the equivalent of 1.5 x 10^6^ infectious units (IU) per well mixed 1:1 with wt9C12 and mut9C12 at 50 μg/ml. AdV DNA was measured from cell lysates produced using lysis buffer (without DNase) from the Taqman Cells-to-CT (Fast) kit (Life Technologies), followed by qPCR for GFP (primers and probe as previously described [2]), using a GFP plasmid standard curve for copy number determination. To compare GFP genome copy number between untreated and PFA-crosslinked virus we used standard curves created from serial dilution of equivalent volumes of AdV and PFA-AdV (S6 Fig).

### Inhibitors

Where stated, 5Z-7-oxozeaenol (0.5 μM, Sigma), panepoxydone (2 μg/ml, Enzo Life Sciences, Farmingdale, NY), epoxomicin (2 μM, Sigma), or DMSO (Sigma) solvent control were added to cells 1 hour prior to infection.

### Confocal microscopy

MEF cells were infected with BrdU-AdV or BrdU-AdV pre-incubated with antibody (as above) for 2 hours. HeLa cells were transfected with FLAG-cGAS (human, OriGene, Rockville, MD) using FuGENE 6 (Promega, Brentwood, UK) 24 hours, before infection as above. Cells were fixed in 4% paraformaldehyde and permeabilized with 0.01% digitonin (Sigma), and non-specific binding was blocked using 5% BSA. BrdU was visualized using BU1/75 (Life Technologies) and AlexaFluor 488-anti-rat (Life Technologies). EdU genome labelling was performed using Click-iT EdU AlexaFluor 488 imaging kit (ThermoFisher Scientific) according to manufacturer’s instructions before staining with AlexaFluor 568-conjugated 9C12. FLAG-cGAS was visualized using M2 anti-FLAG (Sigma) and AlexaFluor 568-anti-mouse. Samples were mounted in Vectashield with DAPI (Vector Laboratories, Peterborough, UK), and imaged using an LSM 780 microscope (Carl Zeiss MicroImaging, Oberkochen, Germany).

### Immunoblotting

Cell lysates were electrophoresed on Nu-PAGE gels (Life Technologies) and transferred to nitrocellulose membranes using the iBlot rapid transfer system (Life Technologies). Antibodies to human TRIM21 (Sc-25351, Santa Cruz) with HRP-conjugated anti-mouse (A0168, Sigma), and HRP-conjugated beta-Actin (Sc-47778, Santa Cruz) were used to detect protein levels.

### DNase activity assay

The DNase activity assay was adapted from the DNase Alert quality control (QC) checking kit (Life Technologies). Cell lysate was prepared from 1 x 10^5^ cells resuspended in hypotonic buffer (20 mM HEPES pH7.5, 5 mM NaF, 10 μM Na_2_MoO_4_ and 100 μM EDTA) and incubated on ice for 15 minutes before addition of 50 μl 10% Nonidet P-40 (New England Biolabs (UK), Ipswich, UK) and vortexing for 10 seconds. Lysates were spun at 13000 xg for 30 seconds at 4°C and the supernatant was collected as the cytoplasmic fraction. Cytosolic extract was mixed with NucleaseAlert buffer and DNA oligonucleotide probe, and cleavage kinetics were monitored using a BMG Pherastar FS platereader at 530/580 nm. Relative fluorescence was calculated by subtracting fluorescence from that achieved by hypotonic buffer and Nonidet P-40 treated controls.

### Cytokine analysis from brain tissue

80 mg sections of forebrain were cut and homogenised in 1 ml Qiazol (Qiagen) using a TissueRuptor (Qiagen). Total RNA was purified using the RNeasy lipid tissue kit (Qiagen) with on-column DNase I digestion kit (Qiagen) according to the manufacturer’s protocol. cDNA was generated from 1 μg purified RNA per reaction, using SuperScript III (200 U per reaction, Life Technologies) and oligo(dT)_23_ primers (7 μM final concentration, Sigma). Relative gene expression was determined as for cDNA from cultured cells (see above).

### Graphs and statistics

Data was graphed using Graphpad Prism 6 software (Graphpad Software, La Jolla, CA). Error bars show standard errors of the mean (SEM). Statistical significance was determined using unpaired two-tailed student’s T-tests, also using Graphpad Prism 6 software.

### Ethics statement

Mouse infection was carried out under project license 80/2534, which was approved by the Medical Research Council Animal Welfare and Ethical Review Body. All experiments adhered to the 19.b.7 moderate severity limit protocol as per the UK Home Office Animals (Scientific Procedures) Act (1986).

## Supporting Information

S1 Fig TRIM21 is required for efficient neutralization of HRV. (**A**) Viability of HeLa cells 7 days post infection with HRV pre-incubated with human serum IgG (IgG), following pre-treatment of cells with recombinant IFNα as indicated. (**B**) As (A), except HeLa cells stably depleted of TRIM21 using shRNA (shT21). (**C**) Viability of TRIM21+/+ and TRIM21-/- MEF cells 7 days post infection with HRV.
(TIFF)

S2 Fig Transcriptional profiles induced by AdV and HRV in the presence and absence of antibody. Fold change in immune transcripts 4 or 8 hours post-infection.
(TIFF)

S3 Fig DNase activity in HeLa cell cytosolic extract. Relative fluorescence generated from cleavage of DNA probe following incubation with HeLa cell cytosolic extract.
(TIFF)

S4 Fig Characterization of mut9C12. **(A)** Hexon binding ELISA **(B)** Anisotropy curves titrating antibody into Alexa488-labelled TRIM21 PRYSPRY.
(TIFF)

S5 Fig siRNA depletion of components of cytosolic nucleic acid sensing pathways. (**A**) Relative STING and MAVS mRNA levels in MEF cells 2 days post transfection with negative control scrambled sequence siRNA (NC si, black), or siRNA directed against MAVS (si MAVS, white) or STING (si STING, gray). (**B**) TNFα mRNA levels 4 hours post transfection of DNA or p(I:C) onto MEF cells treated as in (A). (**C**) Relative RIG-I mRNA levels in MEF cells 2 days post transfection with NC si (black), or siRNA directed against RIG-I (si RIG-I, gray checks). (**D**) TNFα mRNA levels 4 hours post transfection of p(I:C) or p(dA-dT) DNA onto MEF cells treated as in (C). (**E**) Relative cGAS mRNA levels in MEF cells 2 days post transfection with NC si (black), or siRNA directed against cGAS (si cGAS, gray). (**F**) TNFα mRNA levels 4 hours post transfection of DNA onto MEF cells treated as in (E).
(TIFF)

S6 Fig Titration of UT or PFA AdV. Relative detection of GFP gene from UT or PFA treated AdV.
(TIFF)
