# Supplementary figures and images for: TRIM21 Promotes cGAS and RIG-I Sensing of Viral Genomes during Infection by Antibody-Opsonized Virus
Source: PLoS Pathog. 2015 Oct 27;11(10):e1005253. doi: 10.1371/journal.ppat.1005253 (PMC4624778; doi:10.1371/journal.ppat.1005253)

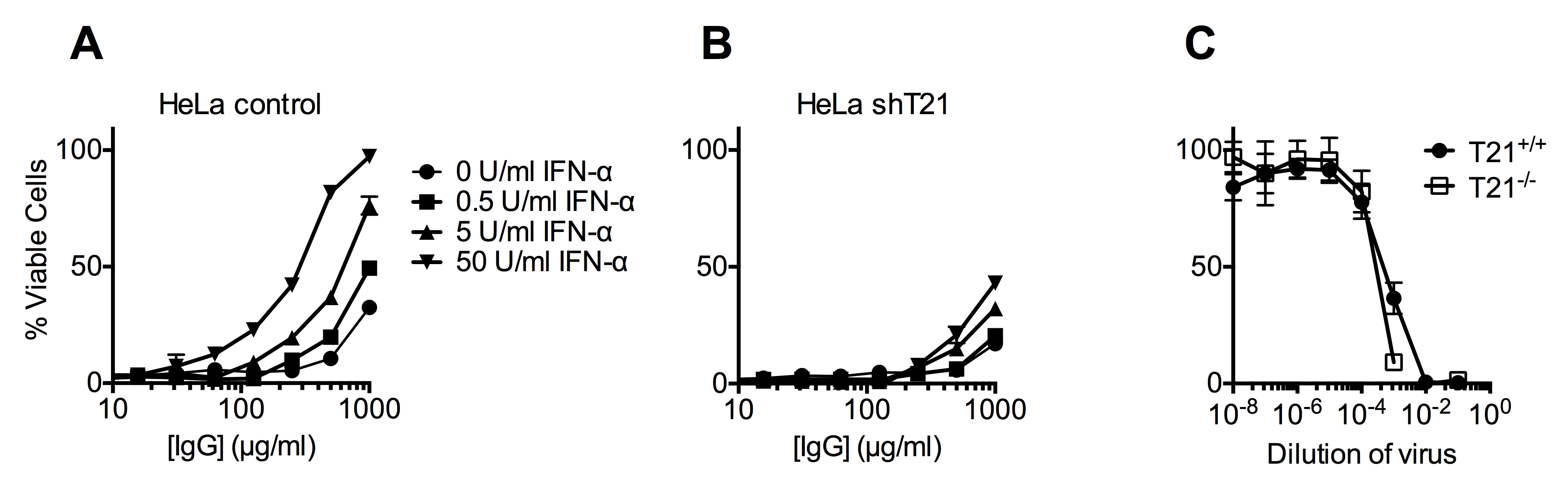

Supplement: S1 Fig — (A) Viability of HeLa cells 7 days post infection with HRV pre-incubated with human serum IgG (IgG), following pre-treatment of cells with recombinant IFNα as indicated. (B) As (A), except HeLa cells stably depleted of TRIM21 using shRNA (shT21). (C) Viability of TRIM21+/+ and TRIM21-/- MEF cells 7 days post infection with HRV. (TIFF) [file ppat.1005253.s001.tiff]

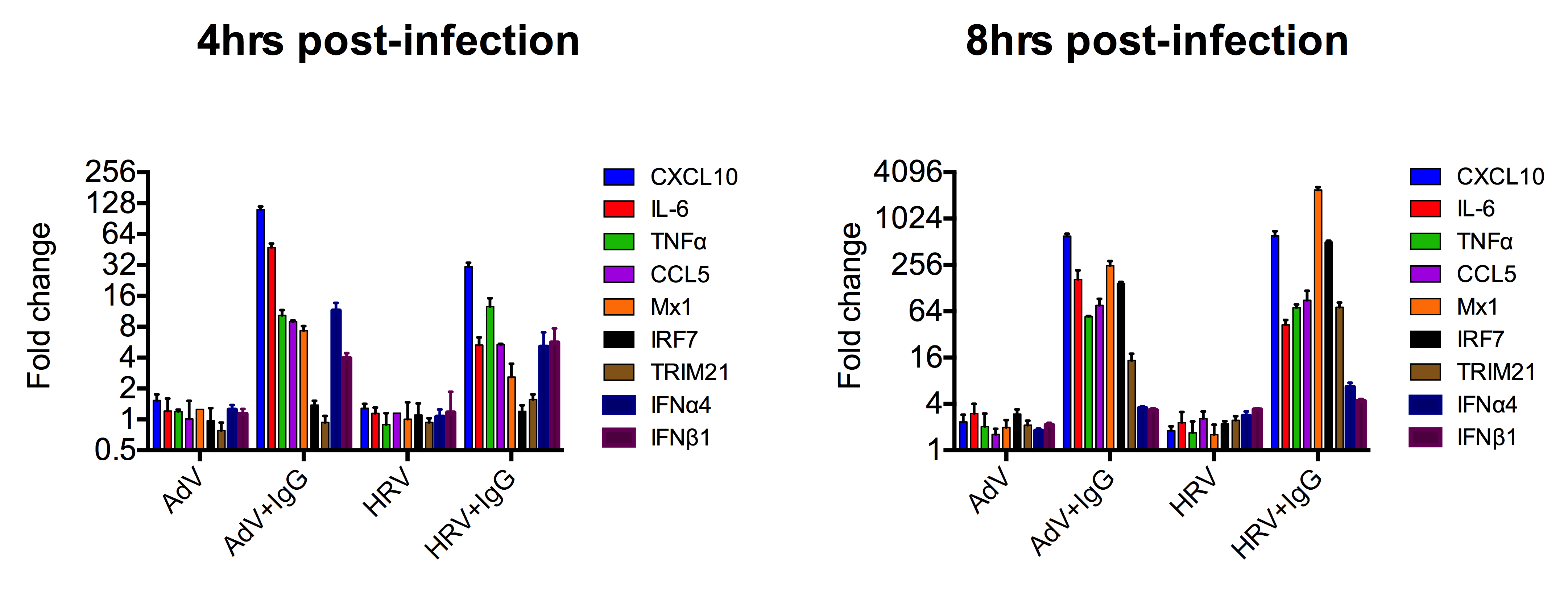

Supplement: S2 Fig — Fold change in immune transcripts 4 or 8 hours post-infection. (TIFF) [file ppat.1005253.s002.tiff]

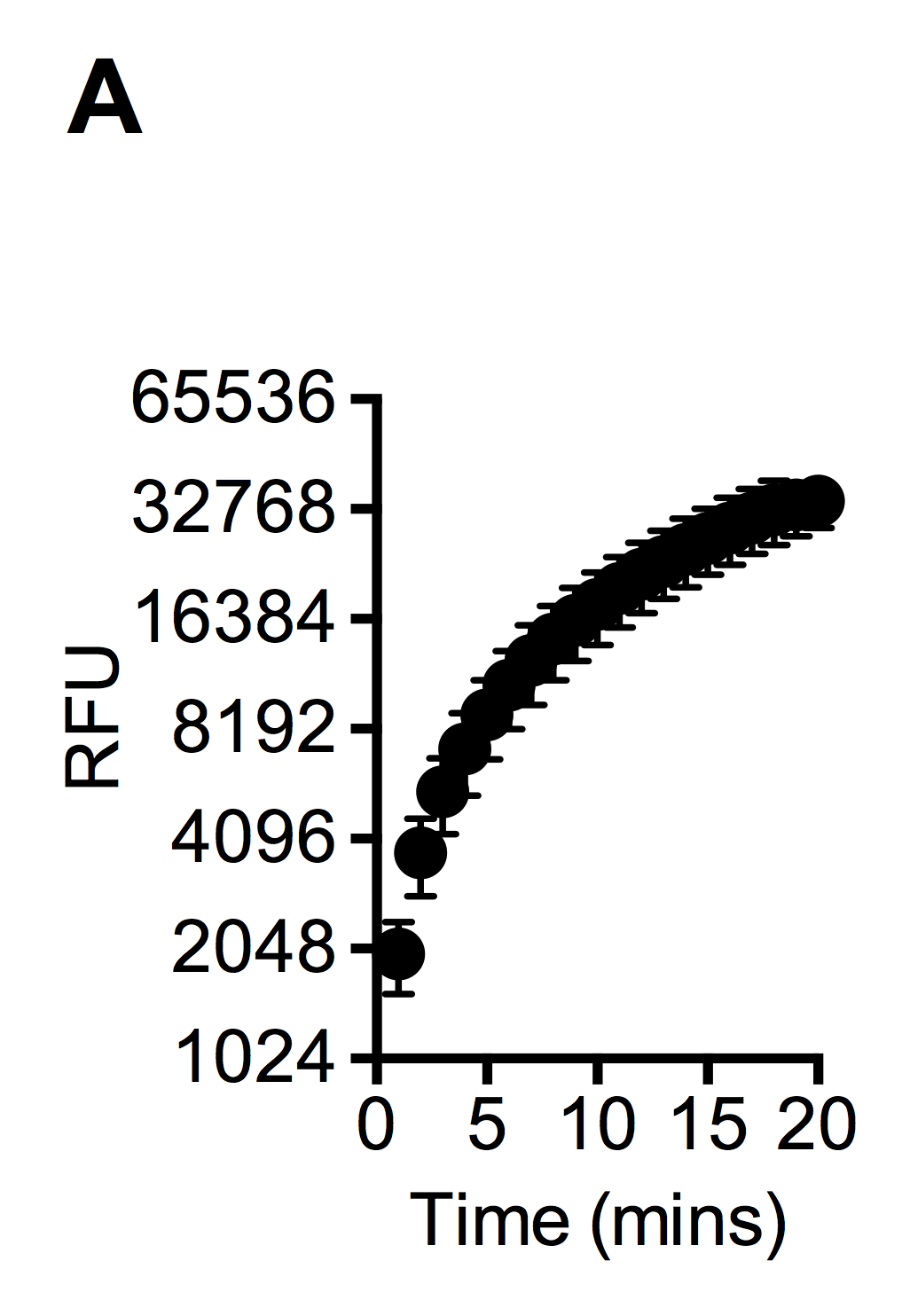

Supplement: S3 Fig — Relative fluorescence generated from cleavage of DNA probe following incubation with HeLa cell cytosolic extract. (TIFF) [file ppat.1005253.s003.tiff]

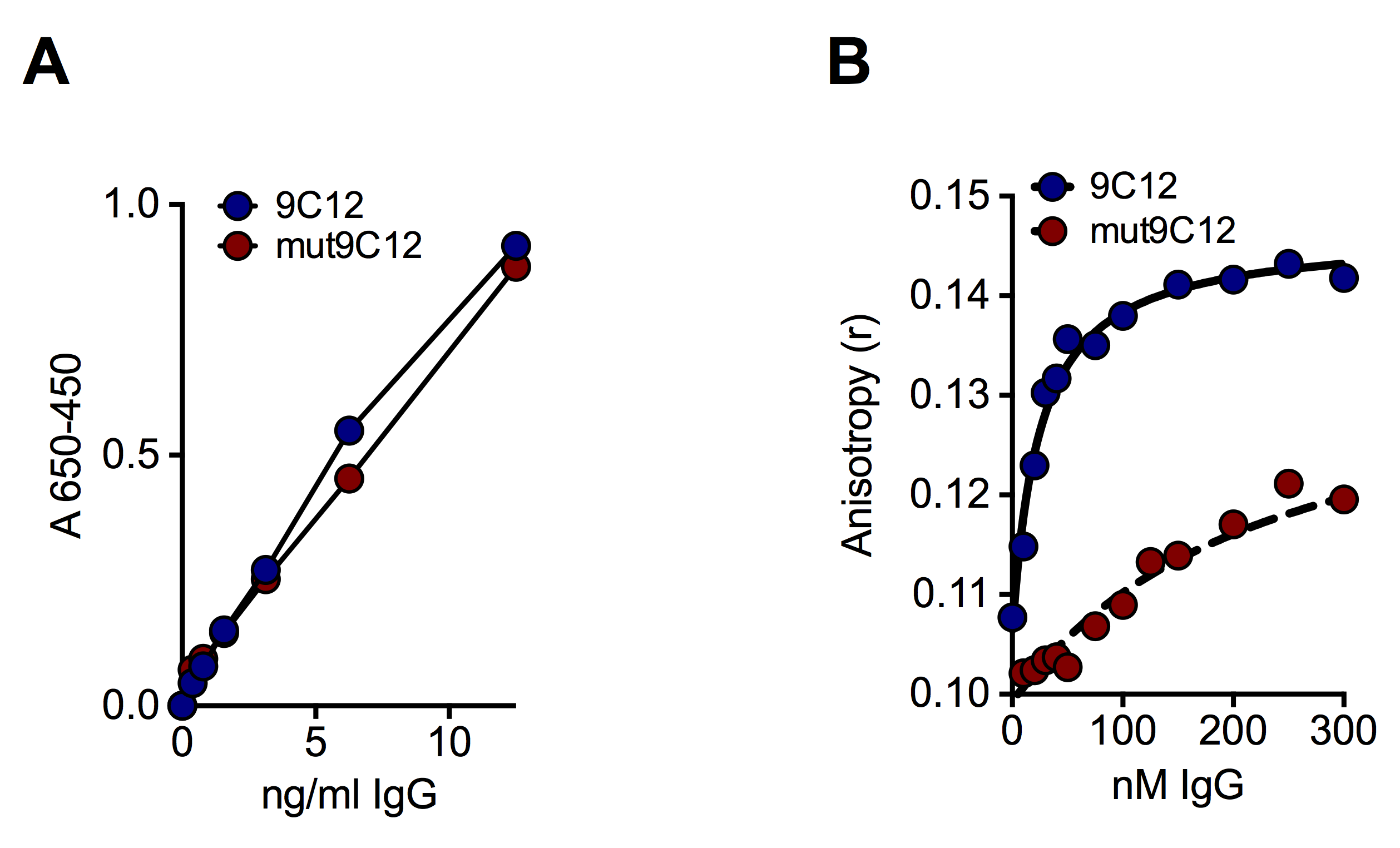

Supplement: S4 Fig — (A) Hexon binding ELISA (B) Anisotropy curves titrating antibody into Alexa488-labelled TRIM21 PRYSPRY. (TIFF) [file ppat.1005253.s004.tiff]

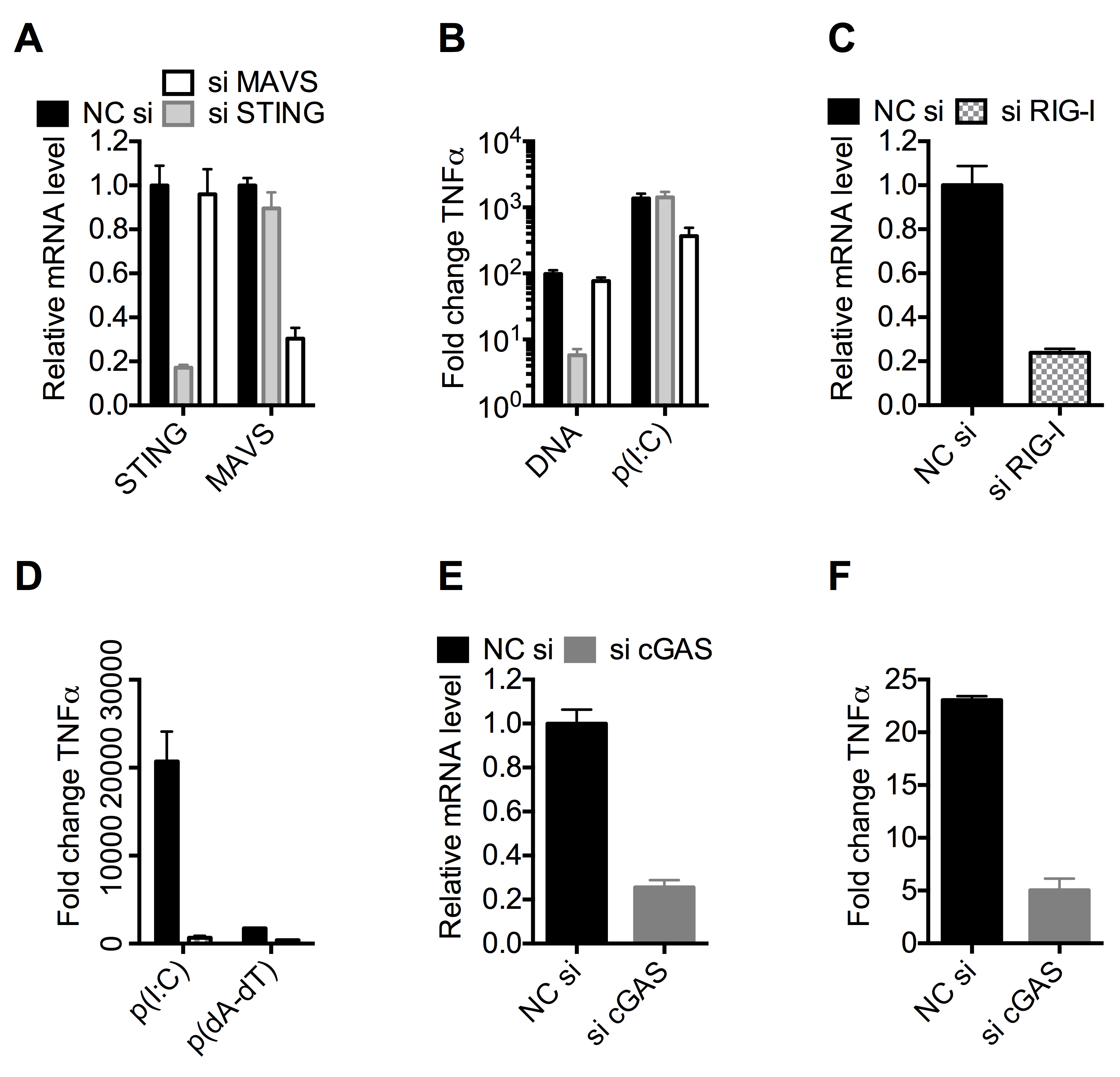

Supplement: S5 Fig — (A) Relative STING and MAVS mRNA levels in MEF cells 2 days post transfection with negative control scrambled sequence siRNA (NC si, black), or siRNA directed against MAVS (si MAVS, white) or STING (si STING, gray). (B) TNFα mRNA levels 4 hours post transfection of DNA or p(I:C) onto MEF cells treated as in (A). (C) Relative RIG-I mRNA levels in MEF cells 2 days post transfection with NC si (black), or siRNA directed against RIG-I (si RIG-I, gray checks). (D) TNFα mRNA levels 4 hours post transfection of p(I:C) or p(dA-dT) DNA onto MEF cells treated as in (C). (E) Relative cGAS mRNA levels in MEF cells 2 days post transfection with NC si (black), or siRNA directed against cGAS (si cGAS, gray). (F) TNFα mRNA levels 4 hours post transfection of DNA onto MEF cells treated as in (E). (TIFF) [file ppat.1005253.s005.tiff]

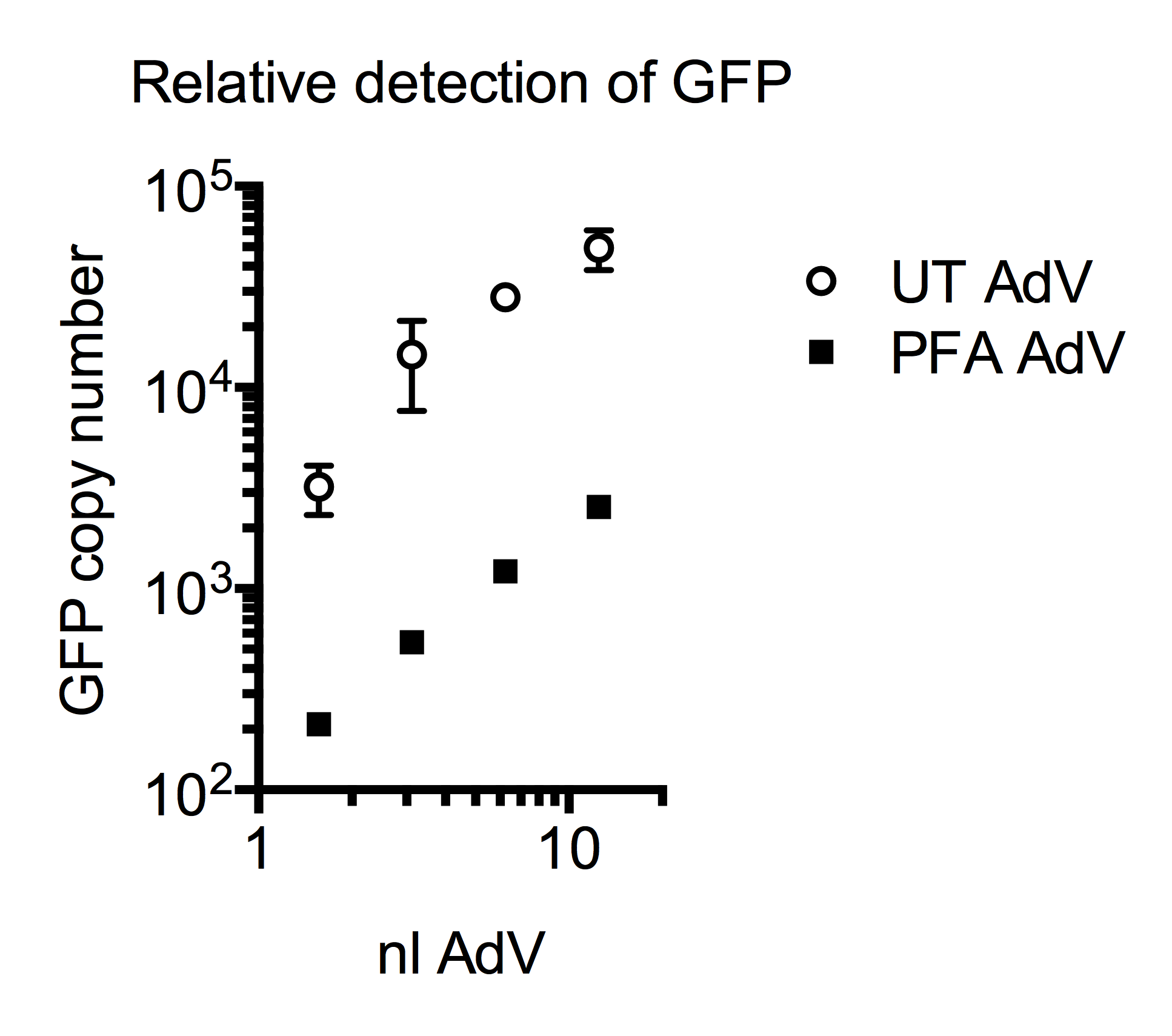

Supplement: S6 Fig — Relative detection of GFP gene from UT or PFA treated AdV. (TIFF) [file ppat.1005253.s006.tiff]
